# Supplementary material for: Superspreading and the evolution of virulence
Source: PLoS Comput Biol. 2025 Oct 9;21(10):e1013517. doi: 10.1371/journal.pcbi.1013517 (PMC12510586; doi:10.1371/journal.pcbi.1013517)
Supplement: S1 Text — (PDF) [file pcbi.1013517.s001.pdf]

Supplementary Information
Superspreading and the evolution of virulence

S1 Adaptive dynamics analysis when transmission is independent of other host characteristics

When transmission is independent of other host characteristics the host contribution to virulence $h(c_i) = 1$ and host natural death rate $d(c_i) = d = 1$ are constant for all host types. The SI version of the model (Equations 1 in the main text) becomes:

$$\frac{dS_i}{dt} = N\left(b - qN\right)p_i - \beta c_i S_i \sum_j c_j I_j - dS_i,$$
$$\frac{dI_i}{dt} = \beta c_i S_i \sum_j c_j I_j - \left(d + \alpha\right) I_i. \tag{S1}$$

We use adaptive dynamics to explore the effect of transmission structure of the host on the evolution of virulence, α. Adaptive dynamics assumes a separation of epidemiological and evolutionary time scales such that the epidemiological dynamics have reached an endemic steady state before a new mutation is considered. When a mutation occurs a mutant strain, with small phenotypic variation from the resident strain, is rare and attempts to invade the resident system at its endemic steady state [1, 2]. To assess how virulence will evolve we derive the fitness function for a mutant strain of infection and determine conditions that allow the mutant to invade the resident population.

We consider a mutant strain of the pathogen with parameters α_M and β_M that attempts to invade a resident pathogen strain with parameters α_R and β_R. The fitness, r_M, of the mutant strain can be determined from the spectral radius (largest eigenvalue) of the mutant sub-matrix of the Jacobian matrix evaluated at the mutant-free, endemic, steady state [3]. To provide an explicit description of how the fitness function is calculated let us consider $n_c = 2$ host types, with transmission levels c_1 and c_2 and probabilities of being born with these transmission levels p_1 and p_2, respectively, and where $p_1 + p_2 = 1$ and $c_1 p_1 + c_2 p_2 = \bar{c}$. The resident and mutant dynamics are

as follows:

$$
\begin{aligned}
\frac{dS_1}{dt} &= N\left(b-qN\right)p_1 - \beta c_1 S_1 (c_1 I_{1R} + c_2 I_{2R} + c_1 I_{1M} + c_2 I_{2M}) - dS_1,\\
\frac{dS_2}{dt} &= N\left(b-qN\right)p_2 - \beta c_2 S_2 (c_1 I_{1R} + c_2 I_{2R} + c_1 I_{1M} + c_2 I_{2M}) - dS_2,\\
\frac{dI_{1R}}{dt} &= \beta_R c_1 S_1 (c_1 I_{1R} + c_2 I_{2R}) - \left(d+\alpha_R\right) I_{1R},\\
\frac{dI_{2R}}{dt} &= \beta_R c_2 S_2 (c_1 I_{1R} + c_2 I_{2R}) - \left(d+\alpha_R\right) I_{2R},\\
\frac{dI_{1M}}{dt} &= \beta_M c_1 S_1 (c_1 I_{1M} + c_2 I_{2M}) - \left(d+\alpha_M\right) I_{1M},\\
\frac{dI_{2M}}{dt} &= \beta_M c_2 S_2 (c_1 I_{1M} + c_2 I_{2M}) - \left(d+\alpha_M\right) I_{2M},
\end{aligned} \tag{S2}
$$

where I_{1R}, I_{2R} denote the density of hosts infected with the resident pathogen strain and with transmission levels c_1 and c_2, respectively, and I_{1M}, I_{2M} denote the density of hosts infected with the mutant pathogen strain and with transmission levels c_1 and c_2, respectively. The mutant strain sub-matrix of the Jacobian, J_{mut}, evaluated at the mutant-free, endemic, steady state is as follows:

$$
J_{mut} = \begin{pmatrix} \beta_M (c_1)^2 S_1 - \alpha_M - d & \beta_M c_1 c_2 S_1 \\ \beta_M c_1 c_2 S_2 & \beta_M (c_2)^2 S_2 - \alpha_M - d \end{pmatrix}, \tag{S3}
$$

where here S_1 and S_2 represent the susceptible density at the mutant-free, endemic, steady state. The eigenvalues of this matrix are given by $\lambda_{1,2}$ as follows:

$$
\lambda_1 = -d - \alpha_M, \qquad \lambda_2 = \beta_M (c_1)^2 S_1 + \beta_M (c_2)^2 S_2 - \alpha_M - d.
$$

As $\lambda_2 > \lambda_1$, the spectral radius, and therefore fitness expression (r_M) for the mutant pathogen strain, is represented by λ_2.

This method for determining the mutant pathogen fitness expression can extend to cases with more than two transmission levels ($n_c > 2$). The fitness of the mutant pathogen strain, r_M, is given by the following expression:

$$
r_M = f(\alpha_M) \sum_{i=1}^{n_c} c_i^2 S_i - (\alpha_M + d). \tag{S4}
$$

Here S_i represents the steady state density of susceptible host type i for the resident and $f(\alpha_M) = \beta_M$. By definition, the fitness, r_R, of the resident population is zero, and therefore

$$
\sum_{i=1}^{n_c} c_i^2 S_i = \frac{\alpha_R + d}{f(\alpha_R)}. \tag{S5}
$$

Using Equation (S5) we can rewrite Equation (S4) to show that the fitness of the mutant strain is positive, $r_M > 0$, if the following condition is satisfied:

$$\frac{f(\alpha_M)}{(\alpha_M + d)} > \frac{f(\alpha_R)}{(\alpha_R + d)}. \tag{S6}$$

Therefore, any invading mutant strain that satisfies Equation (S6) will replace the resident strain and the pathogen evolves a level of virulence, α^*, that maximizes $f(\alpha)/(\alpha + d)$, which is the optimal strategy [4]. The evolutionary singular strategy therefore satisfies the following condition:

$$f'(\alpha^*) = \frac{f(\alpha^*)}{\alpha^* + d}. \tag{S7}$$

This is independent of the transmission level of the host and so when $h(c_i) = 1$ and $d(c_i) = d$ pathogen virulence will evolve to an evolutionarily singular strategy (ESS) at α^* for all transmission distributions of the host.

S1.1 Other model frameworks

Under the same assumptions, other model structures ($SIS, SIR, SIRS$) also lead to the finding that α^* is independent of the transmission level of the host. We specifically show the analysis for the $SIRS$ model framework as this encompasses the other model frameworks. The modification of Equations (1) to represent an $SIRS$ epidemiological framework is as follows:

$$\begin{aligned}
\frac{dS_i}{dt} &= N\left(b - qN\right)p_i - \beta c_i S_i \sum_j c_j I_j - dS_i + \nu R_i, \\
\frac{dI_i}{dt} &= \beta c_i S_i \sum_j c_j I_j - \left(d + \alpha + \gamma\right) I_i, \\
\frac{dR_i}{dt} &= \gamma I_i - dR_i - \nu R_i,
\end{aligned} \tag{S8}$$

where γ is the rate of recovery of infected individuals to the immune class, R, and ν is the rate at which individuals lose immunity and become susceptible once again. We again consider a mutant strain of the pathogen with parameters α_M and β_M that attempts to invade a resident pathogen strain with parameters α_R and β_R. The fitness, r_M, of the mutant strain can be determined as follows:

$$r_M = f(\alpha_M) \sum_{i=1}^{n_c} c_i^2 S_i - (\alpha_M + d + \gamma), \tag{S9}$$

where S_i represents the steady state density of susceptible host type i for the resident. By definition, the fitness, r_R, of the resident population is zero, and therefore

$$\sum_{i=1}^{n_c} c_i^2 S_i = \frac{\alpha_R + d + \gamma}{f(\alpha_R)}. \tag{S10}$$

Using Equation (S10) we can rewrite Equation (S9) to show that $r_M > 0$ if the following expression holds.

$$\frac{f(\alpha_M)}{\alpha_M + d + \gamma} > \frac{f(\alpha_R)}{(\alpha_R + d + \gamma)}. \tag{S11}$$

Therefore, any invading mutant strain that satisfies Equation (S11) will replace the resident strain and the pathogen evolves a level of virulence, α^*, that maximizes $f(\alpha)/(\alpha + d + \gamma)$, which is the optimal strategy. Again, this is independent of the transmission level of the host and so pathogen virulence will evolve to an ESS at α^* for all transmission distributions of the host.

By extending our SI model to an $SIRS$ model framework, we highlight how our results, that increased heterogeneity in transmission leads to a reduced rate of evolution to α^* and increased variation in virulence around α^*, still hold (S1 Fig).

S1.2 Frequency-dependent transmission

The results we have considered so far assume density-dependent infection transmission but our findings extend to frameworks that consider frequency-dependent infection transmission. Again, we show the analysis for the $SIRS$ model framework as this encompasses the other model frameworks. The $SIRS$ epidemiological framework with frequency-dependent transmission is as follows:

$$\begin{aligned}
\frac{dS_i}{dt} &= N\left(b - qN\right)p_i - \frac{\beta}{N} c_i S_i \sum_j c_j I_j - dS_i + \nu R_i, \\
\frac{dI_i}{dt} &= \frac{\beta}{N} c_i S_i \sum_j c_j I_j - \left(d + \alpha + \gamma\right) I_i, \\
\frac{dR_i}{dt} &= \gamma I_i - dR_i - \nu R_i.
\end{aligned} \tag{S12}$$

Following the methods outlined previously, the fitness, r_M, of the mutant strain can be determined as follows:

$$r_M = \frac{f(\alpha_M)}{N} \sum_{i=1}^{n_c} c_i^2 S_i - (\alpha_M + d + \gamma), \tag{S13}$$

where S_i represents the steady state density of susceptible host type i for the resident and N is the total population density at the resident endemic steady state (in the absence of the mutant). By

definition, the fitness, r_R, of the resident population is zero, and therefore

$$\frac{1}{N}\sum_{i=1}^{n_c} c_i^2 S_i = \frac{\alpha_R + d + \gamma}{f(\alpha_R)}. \tag{S14}$$

Using Equation (S14) we can rewrite Equation (S13) to show that $r_M > 0$ if the following expression holds.

$$\frac{f(\alpha_M)}{(\alpha_M + d + \gamma)} > \frac{f(\alpha_R)}{(\alpha_R + d + \gamma)}. \tag{S15}$$

Therefore, any invading mutant strain that satisfies Equation (S15) will replace the resident strain and the pathogen evolves a level of virulence, α^*, that maximizes $f(\alpha)/(\alpha + d + \gamma)$, which is the optimal strategy. Again, this is independent of the transmission level of the host and so in the model framework with frequency-dependent transmission pathogen virulence will evolve to an ESS at α^* for all transmission distributions of the host.

S2 Trade-off function

The trade-off function represented by Equation (3) in the main text is shown in S2 Fig.

S3 Deterministic and stochastic simulations

We present further deterministic and stochastic simulations that correspond to the figures in the main text.

S3 Fig relates to Fig 2.

S4 Fig relates to Fig 3.

S5 Fig, S6 Fig, and S7 Fig relate to Fig 4.

S4 Lower levels of pathogen virulence

In the main paper we detail and explain our key findings for a set-up where the underlying level of virulence is high ($\alpha \in [0, 10]$). We confirm our findings for a set-up when the underlying level of virulence is low ($\alpha \in [0, 2]$). We show that our original results, i.e., that increased heterogeneity in transmission leads to a reduced rate of evolution to the ESS and increased variation in virulence around the ESS, still hold (S8 Fig). We also show that our results where ESS virulence changes as the shape parameter for the transmission distribution changes, under scenarios where the transmission

level of the host is correlated with other host characteristics, still hold when the underlying level of virulence is low (see S9 Fig and compare with Fig 3).

In the main text we explain our findings in terms of the underlying biological processes that drive the evolution of virulence. These explanations also hold for the model with a lower range of virulence. This is an exemplar of how the process-based understanding from general models can be extended to a wider range of scenarios.

References

[1] Geritz, S., Kisdi, E., Meszéna, G. & Metz, J. Evolutionary singular strategies and the adaptive growth and branching of the evolutionary tree. *Evol. Ecol.* **12**, 35–57 (1998).

[2] Metz, J., Geritz, S., Meszena, G., Jacobs, F. & Van HeerWaarden, J. Adaptive dynamics: a geometric study of the consequences of nearly faithful reproduction. In Van Strien, S. & Verduyn Lunel, S. (eds.) *Stochastic and Spatial Structures of Dynamical Systems*, 183–231 (North-Holland, 1996).

[3] Hurford, A., Cownden, D. & Day, T. Next-generation tools for evolutionary invasion analyses. *Journal of the Royal Society Interface* **7**, 561–571 (2010).

[4] Meszena, G., Kisdi, E., Dieckmann, U., Geritz, S. & Metz, J. Evolutionary optimisation models and matrix games in the unified perspective of adaptive dynamics. *Selection* **2**, 193–220 (2002).
